# *AtSIBP1*, a Novel BTB Domain-Containing Protein, Positively Regulates Salt Signaling in *Arabidopsis thaliana*

**DOI:** 10.3390/plants8120573

**Published:** 2019-12-05

**Authors:** Xia Wan, Lu Peng, Jie Xiong, Xiaoyi Li, Jianmei Wang, Xufeng Li, Yi Yang

**Affiliations:** Key Laboratory of Bio-Resources and Eco-Environment of Ministry of Education, State Key Laboratory of Hydraulics and Mountain River Engineering, College of Life Sciences, Sichuan University, Chengdu 610065, China; 2017222040074@stu.scu.edu.cn (X.W.); 2019322040033@stu.scu.edu.cn (L.P.); 2016322040029@stu.scu.edu.cn (J.X.); yiendeavor@gmail.com (X.L.); wangjianmei@scu.edu.cn (J.W.); lixufeng0507@gmail.com (X.L.)

**Keywords:** BTB protein, *AtSIBP1*, salt stress, E3 ligases, *Arabidopsis*

## Abstract

Because they are sessile organisms, plants need rapid and finely tuned signaling pathways to adapt to adverse environments, including salt stress. In this study, we identified a gene named *Arabidopsis thaliana* stress-induced BTB protein 1 (*AtSIBP1*), which encodes a nucleus protein with a BTB domain in its C-terminal side and is induced by salt and other stresses. The expression of the β-glucuronidase (*GUS*) gene driven by the *AtSIBP1* promoter was found to be significantly induced in the presence of NaCl. The *sibp1* mutant that lost *AtSIBP1* function was found to be highly sensitive to salt stress and more vulnerable to salt stress than the wild type WT, while the overexpression of *AtSIBP1* transgenic plants exhibited more tolerance to salt stress. According to the DAB staining, the *sibp1* mutant accumulated more reactive oxygen species (ROS) than the WT and *AtSIBP1* overexpression plants after salt stress. In addition, the expression levels of stress-induced marker genes in *AtSIBP1* overexpression plants were markedly higher than those in the WT and *sibp1* mutant plants. Therefore, our results demonstrate that *AtSIBP1* was a positive regulator in salinity responses in *Arabidopsis*.

## 1. Introduction

In nature, plants must cope with different kinds of abiotic stress, such as high temperatures, cold, salt, drought and osmotic stresses; salt stress is one of the most common and severe stresses [1]. High salinity causes retarded plant growth (even death) and reduces agricultural productivity on more than 20% of the cultivated land worldwide [2]. The unfavorable effects of salt on plants are consequences of both water deficits that result in osmotic stress and excess sodium ions on critical biochemical process [3,4]. In order to deal with unfavorable environmental signals, plants show specific changes in gene expression, metabolism, and physiology in response to different environmental stress conditions [5]. 

In *Arabidopsis*, there are more than 1400 genes that are predicted to encode E3 ubiquitin ligase enzymes [6], which can be largely divided into two groups based on their structure: single-subunit E3 ligases and multi-subunit E3 ligases [7]. Among the multi-subunit E3 ligases, cullin-RING E3 ligases (CRLs) have been best characterized to date. Five cullins (cullin 1/2/3a/3b/4) have been identified as components of the CRLs [7,8]. The cullin3a/3b ligase has been identified in the genome of all eukaryotes, and it interacts with ‘‘Bric-a-brac, Tramtrack and broad (BTB)/complex/pox virus and zinc finger’’ (POZ) (hereafter called BTB) domain proteins [9]. The BTB domain, found in eukaryotes and some viruses, is a highly conserved protein motif with a length of around 116 amino acids that folds into five β-sheets and six α-helices [10]. A BTB domain-containing protein acts as the substrate receptor of cullin 3a/3b and facilitates the assembly of cullin 3a/3b into E3 ligase complexes [11]. The BTB superfamily is divided into multiple groups according to their structure, such as BTB-ZF (BTB zinc-finger), BBK (BTB-BACK-kelch), BBP (BTB-BACKPHR), BTB-ankyrin, all of which contain the BTB domain and other domains, while Skp1 (S-phase kinase associated protein 1) and ElonginC contain only BTB domain [12,13].

BTB plays an important role in plant growth, development, stress resistance, protein ubiquitination and degradation, cytoskeletal composition, ion channel, and the regulation of the cell cycle [14,15]. Previous reports have indicated that the *Arabidopsis* NPR1 (non-expressor of pathogenesis related genes 1) protein that contains the BTB domain is a key regulator at the intersection of the ISR (induced systemic resistance) and SAR (systemic acquired resistance) signaling pathways [16,17,18]. Many BTB protein family members in plants are involved in the regulation of hormone-mediated plant-related signaling such as SA (salicylic acid), JA (jasmonic acid), ABA (abscisic acid), GA3 (gibberellin 3). AHT1 (ABA-HYPERSENSITIVE BTB/POZ PROTEIN 1), a BTB-containing protein, negatively regulates the ABA-mediated inhibition of germination in *Arabidopsis* [19]. Six *Arabidopsis* BTB/POZ-MATH (meprin and TRAF homology) proteins (BPMs) have been reported to participate in ABA responses such as germination and stomatal closing [9]. ATHB6, a homeobox-leucine zipper transcription factor, acts as a negative regulator in ABA and is a target of BPMs for ubiquitination and degradation [19]. The disruption of BT2 (BTB and TAZ(Transcription Adaptor putative Zinc finger) domain protein 2) leads to the enhancement of the ABA-mediated inhibition of germination [9]. Therefore, the BTB-containing protein family is involved in many biological processes in plants including abiotic and biotic stress responses. 

Here, we found a novel BTB domain-containing gene called *AtSIBP1* (*Arabidopsis thaliana* stress-induced BTB protein 1) that belongs to the Skp1 or ElonginC families. The expression of *AtSIBP1* was induced by salt and other stresses. The overexpression of *AtSIBP1* exhibited an enhanced tolerance to NaCl, while the *sibp1* mutant was sensitive to NaCl. The expression levels of stress-responsive genes were significantly increased in *AtSIBP1* overexpression lines. Our analysis demonstrates that *AtSIBP1* acts as a positive regulator in salt stress responses. 

## 2. Results

### 2.1. Expression Pattern of AtSIBP1

**Figure 1:**
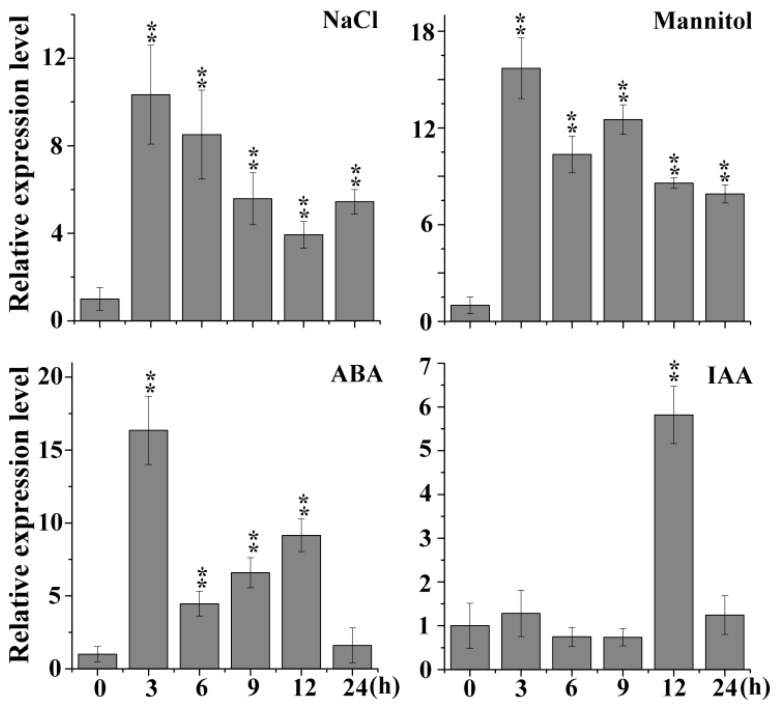
Expression profiles of *AtSIBP1* under stress treatment. An analysis of the expression levels of *AtSIBP1* under abscisic acid (ABA), indole-3-acetic acid (IAA) and abiotic stresses. Eight-day-old wild type (WT) *Arabidopsis* seedlings were treated with 50 μM of ABA, 10 μM of IAA, 200 mM of NaCl and 300 mM of mannitol. The total RNA was extracted at the indicated times. The expression level of *AtSIBP1* was monitored by real-time PCR. *ACTIN2* was used as the internal control. Values are the means (± SD) of three individual experiments, and asterisks indicate significant differences from the WT using the unpaired Student’s t-test (* *p* < 0.05; ** *p* < 0.01).

Most BTB family members are involved in abiotic and biotic stress responses. In the *Arabidopsis* eFP (electronic Fluorescent Pictograph) browser, we noticed a novel gene (*At1g55760*) that is predicted to respond to salt and other stresses and is also a BTB-containing protein (supplementary Figures S1 and S2). Subsequently, the transcripts of *At1g55760* were detected by qRT-PCR under different treatments, and the results showed that the expression levels of *At1g55760* were significantly induced under NaCl, mannitol, ABA and indole-3-acetic acid (IAA) treatment (Figure 1). Therefore, we named it *AtSIBP1* (*Arabidopsis thaliana* stress-induced BTB protein 1). In this study, we focused on the functions of *AtSIBP1* in response to the salt stress.

### 2.2. Identification of Arabidopsis sibp1 Mutant and AtSIBP1 Overexpression Lines

**Figure 2:**
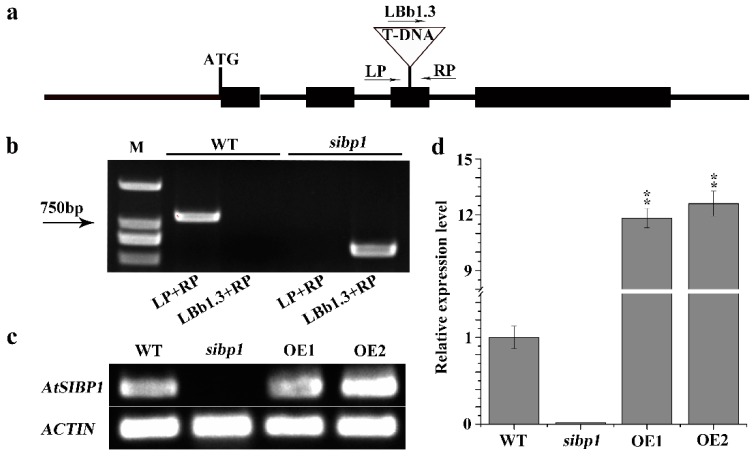
Identification of the *sibp1* T-DNA insertion mutant and construction of *AtSIBP1*-overexpression plants. (**a**) Structure of *Arabidopsis AtSIBP1* and T-DNA insertion site in the *sibp1* mutant (SALK_075267). (**b**) Molecular analysis of *sibp1* and the WT, primers LP, RP and LBb1.3 (Left, Right genomic primer and the left T-DNA border primer) were used to target the flanking sequences of the T-DNA; M represents molecular marker. (**c**) Semi-quantitative RT-PCR analysis of *AtSIBP1* expression in the WT and the *sibp1* mutant. (**d**) qRT-PCR analysis of *AtSIBP1* expression in the WT, the *sibp1* mutant and the *AtSIBP1*-overexpressing lines OE1 and OE2. *ACTIN2* was used as internal control for both semi-quantitative RT-PCR and qRT-PCR.

To investigate the function of *AtSIBP1* in the response of *Arabidopsis* to salt stress, we isolated a homozygous *AtSIBP1* T-DNA insertion mutant (SALK_075267) and generated two independent *AtSIBP1*-overexpression lines (abbreviated as OE1 and OE2). The mutant and the insertion site of *AtSIBP1* was confirmed by PCR and sequencing, respectively (Figure 2a,b). A single copy of T-DNA was inserted into the third exon of *AtSIBP1,* and no full-length *AtSIBP1* transcript was detected in the *sibp1* mutant by RT-PCR analysis, suggesting that *AtSIBP1* is disrupted in *sibp1* mutant plants (Figure 2b). The result of semi-quantitative RT-PCR further confirmed that the transcripts of *AtSIBP1* were totally disrupted in the *sibp1* mutant, while the overexpression of *AtSIBP1* plants increased 11.8 fold in OE1 and 12.6 fold in OE2 compared to the wild type (WT) (Figure 2c,d). 

### 2.3. Tissue Expression Pattern of AtSIBP1 and Subcellular Localization of AtSIBP1-eGFP Fusion Protein

**Figure 3:**
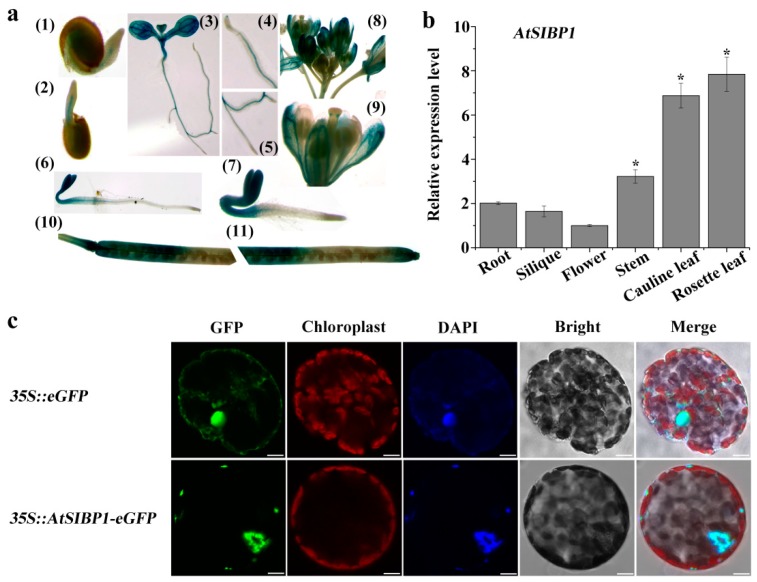
Tissue-specific expression and subcellular localization of *AtSIBP1*. (**a**) Histochemical staining of *proAtSIBP1::GUS* (β-glucuronidase) T3 homozygous plants. *AtSIBP1* promoter activity was detected in the (**1**–**3**) germinating seedlings at day one (1), day two (2), day seven (3), the root tip of the seven-day-old seedlings (4), the lateral root of the seven-day-old seedlings(5), the three-day-old etiolated seedlings (6), the three-day-old light-grown seedlings (7), inflorescence (8), the opened flowers (9), and the siliques (10,11). (**b**) qRT-PCR analysis of *AtSIBP1* expression in different organs. The value for the flower was set to 1. *ACTIN2* was used as an internal control. Values are the means (± SD) of three individual experiments, and asterisks indicate significant differences from the WT using the unpaired Student’s t-test (* *p* < 0.05; ** *p* < 0.01). (**c**) Subcellular localization of *AtSIBP1*-eGFP in tobacco mesophyll protoplasts. At 16 hours after transfection, fluorescence was imaged by confocal microscope. The 35S: eGFP was used as a control. The same cell was stained with DAPI (4’, 6’-diamidino-2-phenylindole) to show its nucleus. Bars = 10 μm.

To further confirm the expression pattern of *AtSIBP1,* the tissue-specific expression of *AtSIBP1* was first analyzed. Transgenic lines carrying *proAtSIBP1::GUS* (β-glucuronidase) were generated, and the T3 homozygous seeds were used for the subsequent staining experiments. Histochemical staining showed that GUS activity could be monitored at all developmental stages (Figure 3a). The GUS staining was first detected in the emerging radicles of the germinated seeds 24h after planting (Figure 3a (1)) and became more obvious in two- and three-day-old seedlings (Figure 3a (2,6,7)). After seven days of growth, GUS activity was found mainly in the leaf and vascular tissues, including the root tips and the lateral roots (Figure 3a (3,4,5)). In mature plants, GUS activity was observed in the generative organs and siliques (Figure 3a (8,9,10,11)). Subsequently, the mRNA of *AtSIBP1* was detected in different organs of *Arabidopsis,* and the results showed that *AtSIBP1* was expressed in all organs; the rosette leaves had the highest expression while the flowers had the lowest expression (Figure 3b). Hence, our results indicate that *AtSIBP1* is expressed throughout the whole life cycle of *Arabidopsis*.

In order to identify the subcellular localization of *AtSIBP1*, a 35S::*AtSIBP1*-eGFP construct was generated and transiently transformed into tobacco mesophyll protoplasts. The green fluorescent signal of the positive control was detected in the nucleus and cytoplasm, while the signal of *AtSIBP1* was only detected in the nucleus (Figure 3c). DAPI (4’, 6’-diamidino-2-phenylindole) dye was used to stain the nucleus, and the results further confirmed that *AtSIBP1* was localized in the nucleus (Figure 3c).

### 2.4. AtSIBP1 Acts as a Positive Regulator in Response to Salt Stress

**Figure 4:**
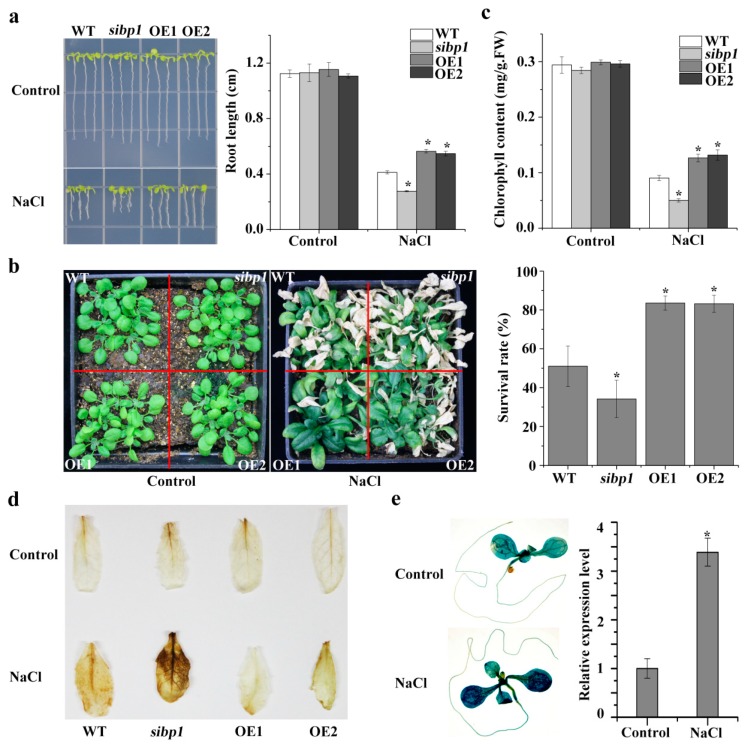
*AtSIBP1* is involved in salt stress response in *Arabidopsis thaliana*. (**a**) Root length of the WT, the *sibp1* mutant, and the overexpression of *AtSIBP1* planted two and a half days on an MS (Murashige and Skoog) medium following a seven-day vertical culture on a 1/2 MS medium supplemented with or without 160 mM of NaCl. (**b**) Phenotype and survival rate of the 16-day-old WT, *sibp1* and *AtSIBP1*-overexpressing lines before and after NaCl treatment. (**c**) Chlorophyll content of the WT, *sibp1,* OE1, and OE2 before and after salt stress. (**d**) DAB staining assay of the rosette leaves of the WT, *sibp1,* OE1, and OE2 with or without salt stress. (**e**) Histochemical staining and expression level of the *GUS* gene in *proAtSIBP1::GUS* T3 homozygous seedlings with or without salt stress. *ACTIN2* was used as an internal control. Values are the means (± SD) of three individual experiments, and asterisks indicate significant differences from the untreated using the unpaired Student’s t-test (* *p* < 0.05; ** *p* < 0.01).

To gain insights into the physiological function of *AtSIBP1* in salt stress, phenotypic experiments were executed. A root growth assay demonstrated that there was no difference among plants under normal conditions (Figure 4a). When plants were exposed to salt stress, the overexpression of *AtSIBP1* plants showed a reduced sensitivity, while the *sibp1* mutant exhibited an increased sensitivity to salt stress compared to the WT (Figure 4a). To further characterize the function of *AtSIBP1* in salt tolerance, the plants grown in soil were also investigated. The overexpression of *AtSIBP1* transgenic plants consistently showed a higher survival rate, while the *sibp1* mutant had a lower survival rate than that of the WT after the treatment with NaCl (Figure 4b). The analysis of chlorophyll contents revealed that the OE1 and OE2 plants had higher chlorophyll contents and that of the *sibp1* mutant was lower than that of the WT after being treated with 200 mM of NaCl (Figure 4c). 

When plants suffer biotic or abiotic stress, the level of reactive oxygen species (ROS) sharply increases and then impairs plant growth [20]. The DAB staining showed that the *sibp1* mutant plants accumulated more ROS (stained deeper), while the OE lines accumulated less ROS after salt stress, and there was no difference in plants under normal conditions (Figure 4d). In addition, the GUS staining of the *Arabidopsis* plants expressing *proAtSIBP1::GUS* also showed that the expression of the *GUS* reporter gene was significantly induced by salt stress (Figure 4e). An analysis by qPCR also revealed an increased level of *GUS* transcripts in *proAtSIBP1::GUS* transgenic *Arabidopsis* under the treatment with NaCl (Figure 4e). Taken together, our results indicate that *AtSIBP1* plays a positive regulator in the salt stress responses of plants.

### 2.5. Disruption or Overexpressing of AtSIBP1 Altered the Expression of Stress Induced Genes

**Figure 5:**
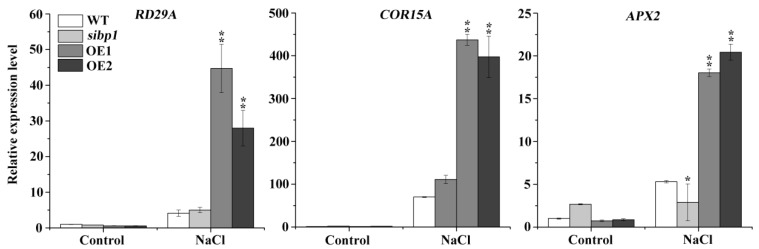
Transcriptional expression levels of stress-inducible genes. Eight-day-old seedlings of the WT, *sibp1,* OE1,and OE2 were treated with 200 mM of NaCl for six hours, and then the transcription levels of *RD29A*, *COR15A* and *APX2* were tested by qRT-PCR. Data represent the means (± SD) from three independent experiments, and asterisks indicate significant differences from the WT using the unpaired Student’s t-test (* *p* < 0.05; ** *p* < 0.01).

To further elucidate the role of the *AtSIBP1* response to salt stress in *Arabidopsis*, eight-day-old seedlings were treated with 200 mM of NaCl for six hours, and then the transcript levels of the stress-inducible genes were analyzed, including the responsive to desiccation 29A (*RD29A*), cold-regulated 15A (*COR15A)*, and ascorbate peroxidase 2 (*APX2)* genes. The expression levels of them were significantly increased in the overexpression of *AtSIBP1* transgenic plants, while the expression level of *APX*2 was decreased in the *sibp1* mutant compared to the WT after the treatment with salt stress (Figure 5). In summary, *AtSIBP1* plays a positive role in plants’ response to salt stress through the up-regulation of the expressions of the stress-inducible genes *RD29A, COR15A* and *APX2*.

## 3. Discussion

In *Arabidopsis thaliana*, there are about 150 BTB proteins that play important roles in the processes of plant growth, development, and stress responses [14,15]. The BTB proteins contain a conserved BTB protein–protein interaction motif and are highly diverse in *Arabidopsis* [21]. In this study, *AtSIBP1* was found to be a typical BTB protein containing one BTB domain and acted as a potential substrate receptor for CRL3 (cullin3-RING E3 ligases) (Supplementary Figure S2).

The analysis of data from the publicly available *Arabidopsis* microarray database and the results of qRT-PCR indicated that *AtSIBP1* was induced by salt stress (Figure 1). In addition, the histochemical staining assay revealed that the *GUS* reporter gene driven by the *AtSIBP1* promoter was expressed in the whole process of the life cycle and was induced by NaCl, suggesting that *AtSIBP1* has an important role in the response to salt stress of plants (Figure 3 and Figure 4e). The root system of plants is one of the most sensitive organs for sensing the availability of water and nutrients or other adverse soil conditions [22,23]. The analysis of root length, seedlings and the content of chlorophyll showed that the *sibp1* mutant was sensitive to NaCl, while the overexpression of *AtSIBP1* transgenic plants were insensitive to NaCl (Figure 4). The mutation of *AHT1*, one BTB-domain protein, resulted in plants that were sensitive to salinity [19]. The overexpression of *AtNPR1,* first the BTB-domain protein cloned in *Arabidopsis*, has been found to have a negative effect on dehydration and salt stresses in rice [24]. However, the overexpression of the *Malus hupehensis MhNPR1* gene has been shown to increase tolerance to salt and osmotic stress by inducing the expression of pathogenesis-related genes (PRs) and osmotic-stress related genes in transgenic tobacco [25]. Abiotic stresses generally increase the production rate of ROS, which may react with proteins, lipids and deoxyribonucleic acid and then cause oxidative damage and impair the normal functions of plant cells [26]. Our DAB staining indicated that the amount of ROS was significantly reduced in the *AtSIBP1* overexpression lines and was accumulated in the *sibp1* mutant (Figure 4d), indicating that *AtSIBP1* is involved in the depression of ROS accumulation in response to salinity.

The nuclear localization of the BTB protein may be a prerequisite for its activation and the regulation of plant immune response [27]. Here, we found that *AtSIBP1* was localized in the nucleus (Figure 3c); thus, we speculate that the degraded target protein is a nucleus-localization regulator in the salt signaling pathway. Our work analyzed the expression levels of salt stress responsive genes, and our results demonstrated that the overexpression of *AtSIBP1* led to the induction of the expressions of *RD29A, COR15A* and *APX2* (Figure 5). Previous reports have indicated that the *RD29A* and *COR15A* promoters contain several DREs (dehydration-responsive elements) and ABREs (abscisic acid-responsive elements), which have the potential to confer abiotic stress resistance. In our study, salt stress induced the expression of *RD29A* and *COR15A* in *AtSIBP1*-overexpressions to increase the tolerance of salinity [28,29]. *APX2*, an ascorbate peroxidase, is one of the most important antioxidant enzymes in plants and detoxifies hydrogen peroxide by using ascorbate as a reductant [30]. The overexpression of *AtSIBP1* was found to result in a higher expression level of *APX2*, indicating an improved antioxidant capacity of plants during salt stress. Therefore, we conclude that the overexpression of *AtSIBP1* leads to increased tolerance of plants to high salinity through the up-regulation of *APX2* expression, and then mediates the reduction of ROS accumulation. 

The functions of BTB proteins in biotic stress have been highly clarified, but their roles in abiotic stress are far from elucidated. In this study, we demonstrated that *AtSIBP1* positively regulated the responses of plants to salt stress. *AtSIBP1*, as a potential substrate receptor for the CRL3 complex, is involved in the tolerance to salt stress. Thus, we infer that its degraded target protein may be a negative regulator in the salt signaling pathway. However, the substrates of *AtSIBP1* have yet to be identified, and the details how *AtSIBP1* works in response to salinity and other stresses need to be further explored in *Arabidopsis*.

## 4. Materials and Methods 

### 4.1. Plant Material and Growth Conditions

All *Arabidopsis* used in this study were of the Columbia (Col-0) ecotype. The T-DNA insertion mutant *At1g55760* (SALK_075267) was obtained from the *Arabidopsis* Biological Resource Center (ABRC). All plants were grown in pots containing a mixture of vermiculite and soil (1:3, *v/v*) in a greenhouse at 22 °C under 70% relative humidity with 16 h light/8 h dark photoperiod. All seeds were sterilized with NaClO (0.5%, *v/v*) for 15min, were washed with sterile water six times, and then stored at 4 °C for 3 d to break dormancy. Surface-sterilized seeds were sown on a Murashige and Skoog (MS) medium containing 2% (*w/v*) sucrose and 0.8% (*w/v*) agar, pH 5.8. 

### 4.2. Generation of Transgenic Plants

To generate the *AtSIBP1*-overexpressing and the *proAtSIBP1::GUS* constructs, the full length CDS (Coding sequence) (990bp) and the promoter of *AtSIBP1* (1521bp) were amplified and cloned into *pCAMBIA1302* and *pCAMBIA1301*, respectively. The constructed plasmids were transformed into *Agrobacterium tumefaciens* (GV3101) and then infiltrated into the WT (wild type) using the floral dip method [31]. The seeds of the transgenic plants were screened on an MS medium supplemented with hygromycin. The mRNA levels of *AtSIBP1* were identified with a qRT-PCR assay. The third generation homozygous seeds of the transgenic plants (T3) were used for further analysis. The primers that were used in this assay were listed in Supplementary Table S1.

### 4.3. Subcellular Localization Assay

The CDS of *AtSIBP1* was cloned into *pBI221* containing a 35S promoter for a green fluorescence protein (eGFP) by homologous recombination. The constructed plasmid 35S::*AtSIBP1*-eGFP and the control of 35S::eGFP were transfected into mesophyll protoplasts from tobacco leaves using the PEG-calcium (polyethylene glycol-calcium) transfection method, as described in [32]. The *pBI221*-eGFP vector was used as a control. To confirm the location of *AtSIBP1*, 4’, 6’-diamidino-2-phenylindole (DAPI) was used to stain the nucleus of the cell. After transfection at 22 °C for 16 h in the dark, the fusion fluorescence proteins were observed and captured using a confocal laser-scanning microscope [33] (Leica TCSSP5 II system, Leica, Germany).

### 4.4. Phenotype Analysis

For the root elongation assay, the seeds were sown on an MS medium for vertical incubation for two and a half days and then transferred to new ½ MS media supplemented with or without NaCl. The root length was determined 1 week after transfer by using the Image J software. For salt treatment, after 16 d, plants grew under normal conditions were irrigated with a 200 mM NaCl solution applied at the bottom of the pots. The morphological phenotypes and survival rates of plants were recorded after 15 d of NaCl treatment. The measurement of chlorophyll content was determined as described [34].

### 4.5. GUS and DAB Staining

The histochemical GUS staining of the homozygous T3 transgenic lines harboring *proAtSIBP1::GUS* was done as described [35]. For DAB (3, 3’-Diaminobenzidine tetrahydrochloride) staining, five *Arabidopsis* rosette leaves treated with NaCl were placed in 5 mL tubes supplemented with a 3 mL DAB staining solution (1 mg/mL DAB, Sigma-Aldrich, adjusted to pH 3.8 with HCl), then vacuumed for 5 min. After 6 h of incubation in the dark with gentle shaking, the leaves were immersed in a bleaching solution (ethanol, acetic acid, and glycerol = 3:1:1) and boiled for 20 min to decolorize the leaves (except for the deep brown polymerization product that was produced by the reaction of DAB with H_2_O_2_), and then the images were captured.

### 4.6. Analysis of Gene Expression

Eight-day-old *Arabidopsis* seedlings were used for gene expression level identification with or without NaCl treatment. The total RNA was extracted from plants with RNAiso Plus reagents (Takara) according to the manufacturer’s instruction. The cDNA was synthesized using 2 μg of RNA by a Prime Script RT reagent Kit with a gDNA Eraser (Takara). For semi-quantitative RT-PCR, the semi-quantitative primers were used to test the expression of *AtSIBP1*. For the qRT-PCR analysis, the TB Green Premix Ex TaqII kit and an Applied Biosystems 7500 real-time PCR system were used for reaction according to the manufacturer’s instruction. *ACTIN2* was used as the internal control. All primers are shown in Supplementary Table S1.

### 4.7. Sequence Analysis of AtSIBP1

The putative protein structure of *AtSIBP1* was obtained from SMART (SIMPLE MODULAR ARCHITECTURE RESEARCH TOOL) and NCBI (National Center for Biotechnology Information). Multiple alignment results were edited in the DNAMAN8.0 program. The phylogenetic analysis was performed with the MEGA7.0 program.

### 4.8. Statistical Analysis

The data are represented as means ± SD (Standard Deviation) (*n* = 3). Statistical analysis was performed using Student’s t-test. The values of *p* < 0.05 were considered to be significant, and Values of *p* < 0.01 were considered as more significant. 

## Supplementary Materials

The following are available online at https://www.mdpi.com/2223-7747/8/12/573/s1. (Figure S1: The development map of *At1g55760* in Arabidopsis eFP browse, Figure S2: Domain organization and phylogenetic analysis of *At1g55760*, Table S1: Primers used in the Paper)
