# Supplementary material for: AtSIBP1, a Novel BTB Domain-Containing Protein, Positively Regulates Salt Signaling in Arabidopsis thaliana
Source: Plants (Basel). 2019 Dec 5;8(12):573. doi: 10.3390/plants8120573 (PMC6963258; doi:10.3390/plants8120573)
Supplement: Supplementary file 1 [file plants-08-00573-s001.pdf]

Article

AtSIBP1, a Novel BTB Domain-Containing Protein, Positively Regulates Salt Signaling in *Arabidopsis thaliana*

Xia Wan, Lu Peng, Jie Xiong, Xiaoyi Li, Jianmei Wang, Xufeng Li and Yi Yang *

Key Laboratory of Bio-Resources and Eco-Environment of Ministry of Education, State Key Laboratory of Hydraulics and Mountain River Engineering, College of Life Sciences, Sichuan University, Chengdu 610065, China; 2017222040074@stu.scu.edu.cn (X.W.); 2019322040033@stu.scu.edu.cn (L.P.); 2016322040029@stu.scu.edu.cn (J.X.); yiendeavor@gmail.com (X.L.); wangjianmei@scu.edu.cn (J.W.); lixufeng0507@gmail.com (X.L.)

* Correspondence: yangyi528@scu.edu.cn; Tel: +86-85412281

Received: 30 October 2019; Accepted: 30 November 2019; Published: date

Legends to Supplementary Figure and Table

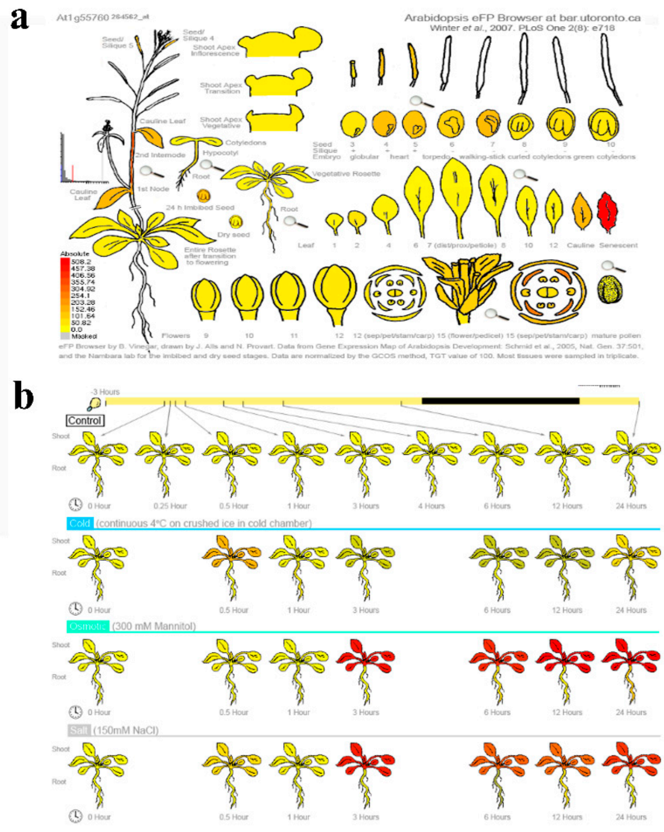

Figure S1 The development map of *At1g55760* in Arabidopsis eFP browser.(a) The development map of *At1g55760*. (b) The predict function of *At1g55760* under salt or other stresses.

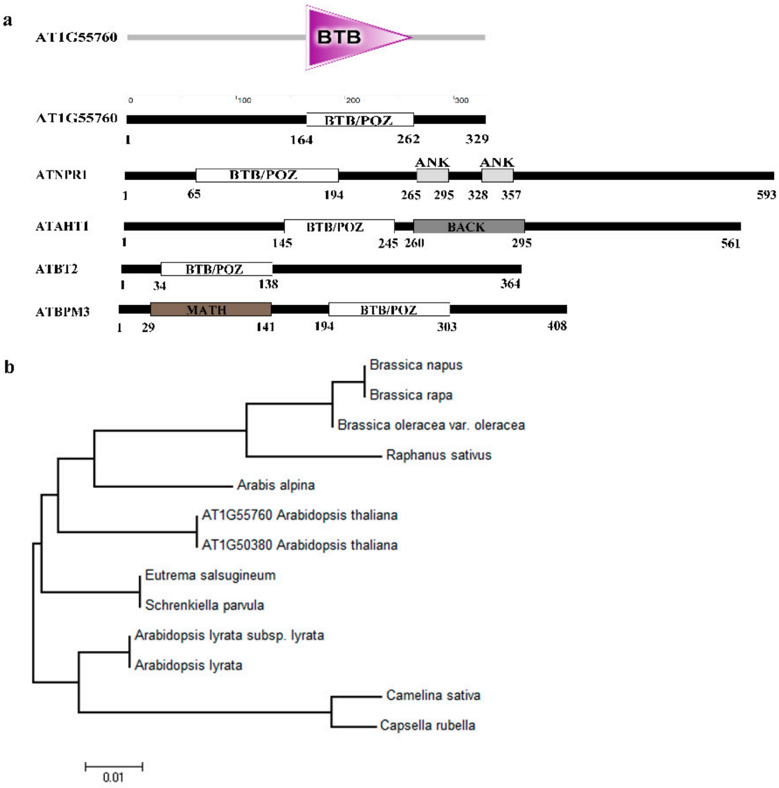

Figure S2. Domain organization and phylogenetic analysis of *At1g55760*. (a) Domain structure of some Arabidopsis BTB/POZ domain proteins. (b) Phylogenetic tree of *At1g55760* protein with its homologs in plants. Phylogenetic analysis was conducted by MEGA7.0.

Table S1. Primers used in the Paper.

primer name	sequence (5' to 3')
AtSIBP1-LP	GAAAATCAGGGGAGAATTTGC
AtSIBP1-RP	TCGTTTTGGATATTGCCGTAG
LBb1.3	ATTTTGCCGATTTCGGAAC
35S-F	GACGCACAATCCCACTATCC
35S-AtSIBP1-F	acgggggactcttgacATGACTGATTCTGCTTACAGAG
35S-AtSIBP1-R	actagtcagatctaccatAAATCCTTTCCAGGTACTGAGG
ProAtSIBP1-GUS-F	ttcgagctcggtacccCAAGATTCCACCAAGACCCC
ProAtSIBP1-GUS-R	taccctcagatctaccatGGCTCTGTAAGCAGAATCAGTCAT
GUS-R	ggacgagtcgtcggttctgt
AtSIBP1-PBI221-F	GAGAACACGGGGGACTCTAGAATGACTGATTCTGCT
AtSIBP1-PBI221-F	ACCACCCGGGGATCCTCTAGAAAATCCTTTCCAGGT
ACTIN-F	ACATCCCACCTACTGGTCTGAAG
ACTIN-R	GCATCTTGGTATTGCTGGTACTCT
AtSIBP1-QRT-F	TTGTATCCAGAGGTATCA
AtSIBP1-QRT-R	AATCCGCTTATCTATTACTT
RD29A-QRT-F	CCAGAAGAAGTTGAACAT
RD29A-QRT-F	CTCGTCATCATCATCATC
COR15A-QRT-F	AAGAGGCATTAGCAGATG
COR15A-QRT-R	GCTTCTTTACCCAATGTATC
APX2-QRT-F	GGCTGGGACATTTGATGTG
APX2-QRT-R	AGGGAACAGCTCCTTGATAGG
